# Vitamin D Is Associated with Clinical Outcomes in Patients with Primary Biliary Cholangitis

**DOI:** 10.3390/nu14040878

**Published:** 2022-02-19

**Authors:** Maryam Ebadi, Stephen Ip, Ellina Lytvyak, Somayyeh Asghari, Elora Rider, Andrew Mason, Aldo J. Montano-Loza

**Affiliations:** 1 Division of Gastroenterology & Liver Unit, Department of Medicine, University of Alberta Hospital, Edmonton, AB T6G 2X8, Canada; ebadi@ualberta.ca (M.E.); sip@ualberta.ca (S.I.); lytvyak@ualberta.ca (E.L.); rider@ualberta.ca (E.R.); andrew.mason@ualberta.ca (A.M.); 2 Department of Clinical Nutrition, Faculty of Nutritional Sciences and Dietetics, Tehran University of Medical Sciences, Tehran 14176-13151, Iran; sasghari@sina.tums.ac.ir

**Keywords:** chronic cholestasis, low vitamin D levels, treatment failure, liver-related mortality, cirrhosis development

## Abstract

Vitamin D (VD) deficiency has been associated with clinical outcomes in patients with chronic liver disease. This study aims to identify the prevalence of VD deficiency in patients with primary biliary cholangitis (PBC) and its association with treatment response to ursodeoxycholic acid (UDCA), cirrhosis development, and liver-related events (mortality and liver transplantation). Two hundred and fifty-five patients with PBC diagnosis were evaluated. Patients with VD levels below 50 nmol/L were defined as deficient. Treatment response to UDCA was defined according to the Toronto criteria. Independent risk factors were identified using binary logistic and Cox regression analysis. The mean level of serum VD was 77 ± 39 nmol/L, and 64 patients (25%) were VD deficient. Incomplete response to UDCA was more prevalent in VD-deficient patients compared to their counterparts (45% vs. 22%; *p* < 0.001). The risk of cirrhosis development (hazard ratio (HR) 1.93; 95% confidence interval (CI) 1.17–3.19, *p* = 0.01) and liver-related mortality or need for liver transplantation (HR 3.33, 95% CI, 1.57–7.07, *p* = 0.002) was higher in VD-deficient patients after adjusting for confounders. Vitamin D deficiency is frequent in patients with PBC and is associated with incomplete response to UDCA, cirrhosis development, and liver-related mortality or need for liver transplantation.

## 1. Introduction

Primary biliary cholangitis (PBC) is a chronic autoimmune cholestatic liver disease. It is associated with biliary epithelial damage and fibrosis development that can lead to cirrhosis, liver failure, and the need for liver transplantation (LT) [1]. PBC has a distinctive autoantibody, the antimitochondrial antibody (AMA), and specific biliary duct histological findings; however, its pathogenesis has not been fully elucidated [2].

Emerging evidence supports the implication of vitamin D (VD) deficiency in the pathogenesis of autoimmune liver diseases [3,4,5]. We demonstrated in patients with autoimmune hepatitis that severe VD deficiency (<25 nmol/L) is related to a higher risk of mortality and disease progression [3]. These findings have engendered interest in VD as a potential factor that can be evaluated, tracked, and intervened in order to improve patients’ outcomes.

Vitamin D is a secosteroid with immune regulatory [6,7,8], antioxidant [9,10], and antifibrotic characteristics [11,12]. Hydroxylation of VD to 25-hydroxyvitamin D (25(OH)D), which is the main circulating form, occurs in the liver [13]. Although this hydroxylated molecule is the key circulating form of VD, it is metabolically inactive. The active metabolite is produced by conversion of 25-hydroxyvitamin D to 1,25-dihydroxyvitamin D in the kidney [13]. Low serum VD levels might mirror liver function failure and insufficient vitamin D3 hydroxylation in chronic liver disease. Other factors that may be associated with vitamin D3 deficiency in various conditions include malabsorption, low sunlight exposure, nutritional deficiency, and impaired cytochrome P450 function [14,15,16].

Lower levels of VD in the serum have been documented frequently in patients with other chronic liver diseases [3,14,17], and severe VD deficiency (≤25 nmol/L) was identified in more than 30% of patients with PBC, which inversely correlated with progression of the disease [4]. Additionally, an association between VD levels and mortality in cirrhosis [18,19], as well as response to ursodeoxycholic acid (UDCA) in PBC [20], has been reported in previous studies. Therefore, VD deficiency might promote liver disease progression in PBC due to the lack of modulation of inflammatory and/or immune-mediated processes.

This study aimed to evaluate the VD serum levels while establishing the prevalence of VD deficiency in North American patients with PBC and identify the association between VD deficiency and insufficient response to UDCA, cirrhosis development, and liver-related events (LT or mortality).

## 2. Materials and Methods

### 2.1. Study Population

A total of 255 white North American patients diagnosed with PBC, according to international guidelines [1,2], were included in this study. All patients were located in Western Canada and assessed in the Autoimmune Liver Disease Clinic at the University of Alberta (Edmonton, Canada) from 1984 to 2019. The Institutional Review Board of the University of Alberta approved this retrospective study (Pro 00089506).

### 2.2. Clinical and Laboratory Evaluations

Liver function tests were assessed periodically at each clinical examination, every 3 to 6 months. Blood level of VD was measured at the time of diagnosis or the first visit to the clinic. This test, which was conducted in the Core Laboratory, University of Alberta Hospital (Edmonton, Canada), determines the level of serum 25(OH)D. Serum VD levels 50 nmol/L [21] were defined as VD deficiency, which aligns with the established cut-off for VD deficiency in patients with liver diseases [19,22,23,24] and other chronic diseases [25]. Serum VD levels between 50 and 75 nmol/L were defined as insufficiency [26].

### 2.3. Diagnosis of Cirrhosis at Baselines or during Follow-Up

The presence of histological cirrhosis at diagnosis or in following liver tissue biopsies, increased FibroScan reading measurement by vibration-controlled transient elastography (>14 kPa), radiological demonstration of cirrhosis by computerized tomography (CT) or magnetic resonance imaging (MRI) (Edmonton, Alberta, Canada), and progression of portal hypertension or its complications indicated cirrhosis at diagnosis or cirrhosis development in patients without this finding at baseline.

### 2.4. Incomplete Response to UDCA Therapy

All patients were treated with UDCA at a dose of 13 to 15 mg/kg per day according to American Association for the Study of Liver Diseases (AASLD) guidance [2] and the European Association for the Study of the Liver (EASL) guidelines [1]. Incomplete responders to UDCA were defined according to the Toronto criteria [27,28].

### 2.5. Liver-Related Events

Liver-related events were defined as LT or liver-related mortality. Liver-related mortality was described as death from liver failure, hepatocellular carcinoma, or complications of portal hypertension such as variceal bleeding, hepatic encephalopathy, or hepatorenal syndrome.

### 2.6. Statistical Analysis

Mean and standard deviation (SD) were used to present continuous variables, and the differences between means were analyzed using the unpaired t-test. Associations between categorical variables were determined using Fisher’s exact test. Vitamin D prognostic implication was initially evaluated as a continuous variable followed by performing comparisons between patients with and without VD deficiency (< and ≥50 nmol/L).

Kaplan–Meier methodology with the log-rank (Mantel–Cox) test was applied to calculate the survival probabilities and determine the significance of differences between survival curves. Patients were censored at the time of their last visit if they were lost during follow-up or were alive at the last follow-up.

Univariate and multivariate Cox proportional hazards regression was used to analyze the association between VD and outcomes, and the results were presented as hazard ratios (HR) with 95% confidence intervals (CI). Factors contributing to incomplete response to UDCA were determined using binary logistic regression analysis. Factors with a *p* < 0.10 in the univariate analysis were incorporated into multivariate models. Statistical significance was determined as a *p* < 0.05 using IBM SPSS Statistic Software 26 (SPSS for Windows, version 26.0, SPSS, Chicago, IL, USA).

## 3. Results

### 3.1. Patient Characteristics

Demographic, clinical, and biochemical characteristics are presented in Table 1. The majority of patients were female (*n* = 223; 88%) with a mean age of 53 ± 12 years at diagnosis. The mean body mass index (BMI) was 27 ± 6 kg/m^2^, and 73% of the patients were overweight or obese (BMI ≥ 25 kg/m^2^).

The mean VD level was 77 ± 39 nmol/L (range, 4–201 nmol/L). Of the 255 patients with PBC, 24 (9%) had severe VD deficiency (<25 nmol/L), while 64 patients (25%) and 65 patients (25%) presented with VD deficiency (<50 nmol/L) and insufficiency (50–70 nmol/L), respectively.

Clinical features of patients with and without VD deficiency (<50 vs. ≥50 nmol/L) are presented in Table 1. Diabetes was more common in patients diagnosed with VD deficiency (25% vs. 12%, *p* = 0.01). Patients with VD deficiency had lower serum albumin levels but higher bilirubin and alkaline phosphatase. The frequency of cirrhosis at diagnosis, cirrhosis complications including ascites and hepatic encephalopathy, UDCA-incomplete response, and liver-related events was higher in patients with VD deficiency. There were no significant differences in age at diagnosis, sex, BMI, or seasonal variation in VD status between groups with different VD statuses.

### 3.2. Treatment Response to UDCA

All patients were treated initially with UDCA, and in 19 patients (8%), second-line treatment was used (obeticholic acid and bezafibrate). At 12 months, 73% had a complete response to UDCA. Patients who had accomplished complete response to UDCA had significantly higher VD levels at presentation compared to those who did not (82 ± 37 vs. 65 ± 41 nmol/L; *p* < 0.001). Incomplete response to UDCA was noticed more frequently in patients with VD deficiency (45% vs. 22%, *p* < 0.001; Table 1).

Serum VD level (nmol/L) as a continuous variable (odds ratio (OR) 0.99, 95% CI, 0.98–0.996, *p* = 0.002) and VD deficiency (OR 3.03, 95% CI, 1.66–5.53, *p* < 0.001) was significantly related to the UDCA-incomplete response in univariate analysis (Table 2). After adjusting for other significant predictors (age at diagnosis and BMI) in multivariate analysis, serum VD levels (OR 0.99, 95% CI, 0.98–0.997, *p* = 0.01) and VD deficiency (OR 2.92, 95% CI, 1.53–5.56, *p* = 0.001; Table 2) were significantly associated with the risk of incomplete response to UDCA.

### 3.3. Development of Cirrhosis

**Figure 1:**
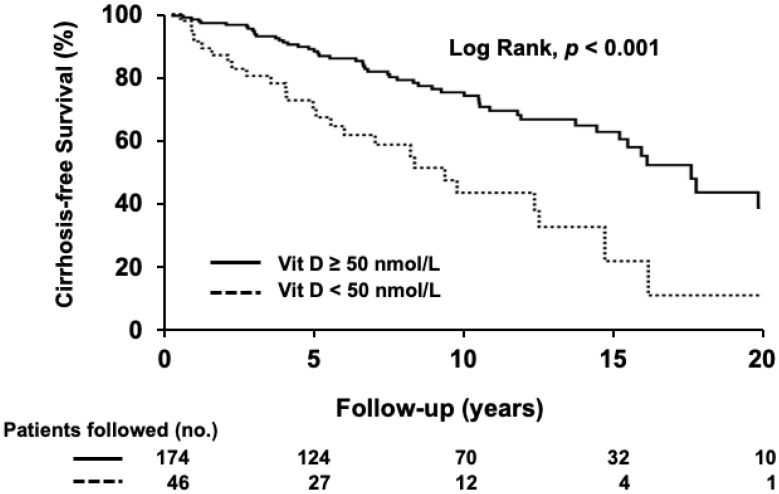
Cirrhosis-free survival in patients with and without vitamin D (VD) deficiency. Survival over time was assessed using Kaplan–Meier curves and the comparison between curves was performed using the log-rank test. Cirrhosis-free survival was shorter in patients with VD deficiency compared to the patients without deficiency (*p* < 0.001). Number of followed patients at each time-point is shown below the figure.

Cirrhosis was present in 35 patients at diagnosis (14%), and among patients without cirrhosis at presentation (*n* = 220), 80 patients (36%) developed cirrhosis during follow-up. Shorter median time to cirrhosis development was observed in patients with VD deficiency (10 years; 95% CI 7–13) when compared to the patients without deficiency (19 years; 95% CI, 16–21; *p* < 0.001; log-rank test, Figure 1).

Serum VD level and VD deficiency were both predictors of progression to cirrhosis in univariate Cox analysis (Table 3). Serum VD levels (HR 0.99, 95% CI, 0.98–0.996, *p* = 0.002) and VD deficiency (HR 1.93, 95% CI, 1.17–3.19, *p* = 0.01) were independently associated with cirrhosis development after adjusting for age at diagnosis, sex, diabetes, and treatment response to UDCA.

### 3.4. Liver-Related Mortality or Liver Transplantation

Among 255 patients, 37 (15%) received LT or died due to liver-related diseases when followed for a median period of 8 years (mean, 9 ± 6 years). The frequency of poor clinical endpoints (LT or liver-related death) was significantly higher in patients with VD deficiency than patients without deficiency (30% vs. 9%, *p* < 0.001; Table 1).

**Figure 2:**
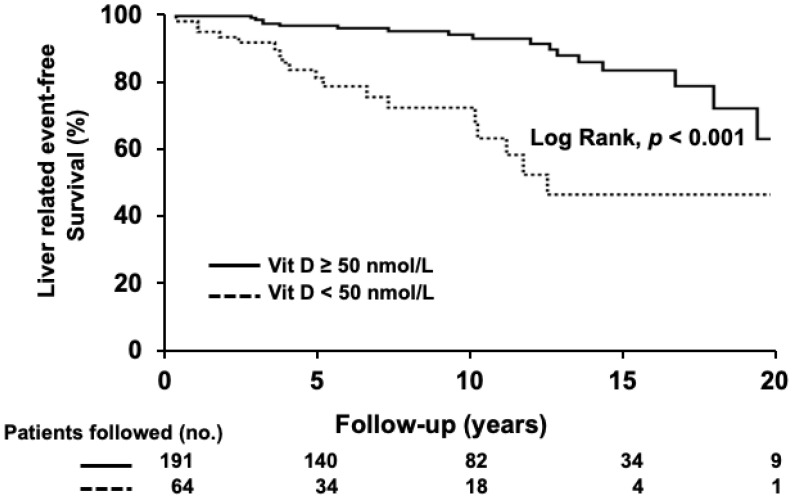
Liver-related event-free survival in patients with and without VD deficiency. Survival over time was assessed using Kaplan–Meier curves and the comparison between curves was performed using the log-rank test. Shorter liver-related event-free survival was noticed in VD-deficient patients compared to those without deficiency (*p* < 0.001). Number of followed patients at each time-point is shown below the figure.

The mean level of serum VD was lower in patients with unfavorable outcomes (52 ± 37 vs. 81 ± 38 nmol/L, *p* < 0.001). Patients with VD deficiency received LT significantly more often than patients without deficiency (20% vs. 6%, *p* = 0.003). The 5- and 10-year probability of liver-related event-free survival was 82% and 73% in patients with VD deficiency and 97% and 95% in patients without deficiency (log-rank, *p* < 0.001, Figure 2).

In univariate analysis, assessing the association between clinical parameters and liver-related events, diabetes, cirrhosis at diagnosis, UDCA-incomplete response, BMI ≥ 25 kg/m^2^, and VD deficiency were associated with less favorable outcomes (Table 4). Each 1 nmol/L increase of serum VD level significantly decreased the risk of liver-related events by 3% (HR 0.97, 95% CI, 0.96–0.98, *p* < 0.001; Table 4). Patients with VD deficiency were 3 times more likely to receive LT or die due to the liver-related diseases over the time of follow-up (HR 3.33, 95% CI, 1.57–7.07, *p* = 0.002, Table 4) after adjusting for diabetes, BMI ≥ 25 kg/m^2^, cirrhosis at diagnosis, and UDCA-incomplete response.

### 3.5. Response to VD Supplementation

Descriptive data presented here are collected from patients’ charts as part of an observational study but not a clinical trial. The mean VD level of all 255 patients was 77 ± 39 nmol/L. Of those, 69 patients were supplemented with VD irrespective of VD status at baseline, and the mean VD level increased to 98 ± 47 nmol/L in supplemented patients (*p* < 0.001). Of 64 VD-deficient patients, 28 patients (44%) were supplemented with VD; however, the dose of supplementation considerably varied between patients including annual intramuscular injection of a megadose of 625,000 or 500,000 international units (IUs), 50,000 IU of vitamin D2, or 6000 to 1000 IUs of D3. The duration at which the dose was provided varied from 1 to 13 months with short- or long-term intervals between doses. Serum VD levels following supplementation were available for only 17 patients, and of them, only one patient had deficiency.

## 4. Discussion

In this study, we found that VD deficiency is present in 25% of patients with PBC at the time of diagnosis and is associated with UDCA-incomplete response and increased need for LT and liver-related death. We also found that not only was cirrhosis at diagnosis more prevalent among VD-deficient patients, but VD deficiency was also associated with a higher risk of cirrhosis development. These findings are consistent with and support those of prior studies showing an increased prevalence of low VD levels in patients with PBC and association between pretreatment VD status with response to UDCA and progressive liver failure [4,20].

Emerging evidence supports the significance of VD deficiency in the pathogenesis of various liver diseases (reviewed in [29,30]). However, it has not been identified whether VD deficiency is a reason for or the result of chronic liver disease. We cannot disregard the probability that VD deficiency was a former unknown feature contributing to the development of PBC prior to its diagnosis. Diminished hydroxylation by hepatocytes, undiagnosed chronic liver disease, low levels of binding proteins (VD binding protein and albumin), increased storage of VD by adipose tissue, reduced intestinal absorption, decreased dietary intake, and limited sun exposure (reviewed in [17]) are factors contributing to low VD levels in chronic liver disease. Nevertheless, there was no association between seasonal variation in sun exposure and VD status in this study, which might be related to the limited sun exposure in this high-latitude part of Canada. Previous studies of VD deficiency in chronic liver disease indicated that impaired hydroxylation of cholecalciferol by the liver rather than sun exposure is a major contributor to VD deficiency in patients with cirrhosis [19] and hepatocellular carcinoma [24]. On the other hand, VD deficiency may serve as a marker of PBC severity, given the higher prevalence of cirrhosis complications and worse liver function tests, reflected as lower albumin, as well as higher bilirubin and alkaline phosphatase, levels in patients with deficiency. Whether liver dysfunction in PBC is a compensatory mechanism for altered VD metabolism and consequently its deficiency, or the VD deficiency that contributes to the progression of liver damage due to its immunomodulatory and anti-inflammatory properties [29], remains uncertain.

Impaired production of 1,25-dihydroxyvitamin D could support the proliferation of immune cells by deteriorating the regulation of the activity of nuclear DNA polymerase and protein kinase [31]. This may impact the stimulation of the *FOXP3* gene and regulatory T cells [32,33], the production of cytotoxic T lymphocyte antigen-4 (CTLA-4) and IL-10 [33], proinflammatory responses induced by toll-like receptors [34], production and transformation of glutathione [9], and oxidative stress [9]. Activation of hepatic stellate cells [35], downregulation of the matrix metalloproteinases activity, and collagen deposition augmentation [35] are other pathways by which VD deficiency may stimulate hepatic fibrosis. Antiproliferative and antifibrotic effects of VD on liver fibrosis are mechanisms by which VD may exert beneficial effects in PBC [36].

Lack of proper regulation of the inflammatory, immune, and fibrotic responses to prolonged liver damage are factors contributing to the association between VD deficiency, treatment incomplete response, and cirrhosis development [35]. Developing liver failure could sequentially decrease vitamin D3 hydroxylation in the liver and result in the need for LT or liver-related mortality. This theory assumes VD deficiency could act as a biomarker and pathogenic factor, indicating the development of liver failure and stimulating homeostatic perturbations, respectively. These observations demonstrate the necessity to examine VD supplementation as a therapeutic intervention in prospective clinical settings. The impact of VD supplementation on the improvement to UDCA response awaits additional research.

The prevalence of VD deficiency is higher in children and older adults, patients with obesity, and patients in high-latitude countries [21,37,38]. Given limited dietary sources, food fortification by VD or biofortification, as well as oral supplementation, has been utilized to improve VD status in the general population [39,40]. The common dose of VD supplementation to raise the serum 25(OH)D by 15–25 nmol/L is 1000 IU; however, recommendations vary across countries in the range of 400 to 2000 IU per day [21]. In the case of VD deficiency or insufficiency, supplementation dose needs to be determined based on the baseline 25(OH)D serum levels and age [41]. Despite the lack of commonly established repletion treatments, both oral and intramuscular cholecalciferol (300,000 IU) effectively increased serum 25(OH)D levels in VD-deficient healthy adults [42]. The efficiency and safety of VD high doses were evaluated in a recent systematic review revealing VD megadoses were effective and convey minimal toxicity [43].

Vitamin D deficiency has an important role in the onset and progression of many chronic diseases such as cancer and autoimmune diseases [44]. In liver diseases, VD-based treatments protect against the progression of the disease by antifibrotic effects [45]. In PBC, VD supplementation may help to moderate the inflammatory response, oxidative stress, and profibrogenic activity, preventing the progression of liver fibrosis [36]. Vitamin D supplementation may also improve treatment response in PBC given its role in the regulation of response to cholestasis and immune stimuli [20]. Association between UDCA-incomplete response and adverse outcomes with VD deficiency in this study demonstrates the necessity to perform prospective clinical trials in order to determine whether VD supplementation can improve outcomes and response to treatment in PBC patients.

In a subgroup analysis of patients who were taking VD supplements, VD levels following supplementation were available for only 17 patients, of whom only one had deficiency. Due to the absence of guidelines regarding vitamin D supplementation in PBC, our data on VD supplementation included variable doses and durations. Improvement in circulating 25-hydroxyvitamin D levels during supplementation may reflect the hepatic efficiency in cholecalciferol hydroxylation in these patients; however, clinical trials evaluating the optimal VD supplementation dose in patients with PBC and potential improvements in prognosis are required.

We acknowledge that our study has several limitations due to its retrospective nature with a relatively small number of patients. The lack of comparative healthy controls does not allow us to make a clear conclusion on the status of VD in PBC patients. The major drawback is that this study cannot conclusively discriminate whether VD deficiency itself results in adverse clinical outcomes or whether it is a marker of PBC severity. Although limited dietary sources of VD demonstrate the need for supplementation in seasons with low sun exposure, dietary intake of VD was not assessed in this study. Genetic polymorphisms in VD metabolism were also not considered [46]. Data on VD supplementation were collected as part of a retrospective chart review, and a clinical trial was not conducted; thus, dose and duration of VD supplementation varied between patients, and confidential results on supplementation could be obtained.

## 5. Conclusions

In conclusion, VD deficiency presents in 25% of patients with PBC and is associated with insufficient response to UDCA and cirrhosis development. A low pretreatment VD serum level also emerged as an independent predictor of liver-related mortality or need for LT. Regular assessment of VD levels should be recommended in patients with PBC, which may serve as a predictive biomarker. Further, it would be important to assess the impact of VD supplementation on disease progression and patients’ outcomes in prospective clinical trials.

## Figures and Tables

**Table 1:** Clinical features associated with vitamin D deficiency in PBC.

Characteristics	All (*n* = 255)	<50 nmol/L (*n* = 64)	≥50 nmol/L (*n* = 191)	*p*-Value
Age at diagnosis, years	53 ± 12	52 ± 13	53 ± 12	0.73
Sex, male	32 (13)	9 (14)	23 (12)	0.67
BMI, kg/m^2^	27 ± 6	26 ± 6	27 ± 5	0.10
BMI ≥ 25 kg/m^2^	186 (73)	42 (66)	144 (75)	0.14
Hypertension	78 (31)	20 (31)	58 (30)	0.88
Diabetes	38 (15)	16 (25)	22 (12)	0.01
Albumin (g/L)	40 ± 5	38 ± 6	41 ± 4	<0.001
Alkaline phosphatase (IU/L)	209 ± 160	299 ± 209	179 ± 127	<0.001
Bilirubin (µmol/L)	14 ± 14	21 ± 23	12 ± 8	0.005
VD (nmol/L)	77 ± 39	30 ± 13	93 ± 31	<0.001
Hepatic encephalopathy	19 (7)	11 (17)	8 (4)	0.002
Ascites	51 (20)	24 (38)	27 (14)	<0.001
Cirrhosis at diagnosis	35 (14)	18 (28)	17 (9)	<0.001
UDCA-incomplete response	70 (28)	29 (45)	41 (22)	<0.001
Development of cirrhosis	80 (31)	25 (54)	55 (32)	0.006
Liver-related events	37 (15)	19 (30)	18 (9)	<0.001
Seasonal variation				
Spring–summer	122 (48)	31 (48)	91 (48)	1.00
Fall–winter	133 (52)	33 (52)	100 (52)	1.00

Abbreviations: VD—vitamin D; PBC—primary biliary cholangitis; BMI—body mass index; UDCA—ursodeoxycholic acid. Numbers in parentheses are percentages. Data are shown as mean ± standard deviation (SD).

**Table 2:** Features related to incomplete response to UDCA.

	Univariate		Multivariate Serum VD Level (nmol/L)		Multivariate (VD Deficiency)	
Characteristics	OR (95% CI)	*p*-Value	OR (95% CI)	*p*-Value	OR (95% CI)	*p*-Value
Age at diagnosis, years	0.97 (0.95–0.99)	0.009	0.97 (0.95–0.99)	0.01	0.97 (0.95–0.99)	0.009
Sex, male	0.57(0.23–1.46)	0.24				
Hypertension	0.65(0.35–1.22)	0.18				
Diabetes	0.79 (0.36–1.77)	0.57				
Cirrhosis at diagnosis	0.76 (0.33–1.75)	0.51				
BMI ≥ 25 kg/m^2^	0.25 (0.14–0.45)	<0.001	0.24 (0.13–0.45)	<0.001	0.25 (0.13–0.46)	<0.001
Serum VD level, nmol/L	0.99 (0.98–0.996)	0.002	0.99 (0.98–0.997)	0.01		
VD deficiency	3.03 (1.66–5.53)	<0.001			2.92 (1.53–5.56)	0.001

Abbreviations: BMI—body mass index; CI—confidence interval; OR—odds ratio; UDCA—ursodeoxycholic acid. Binary logistic regression was applied to estimate ORs and *p*-values.

**Table 3:** Clinical Features Associated with Cirrhosis Development.

	Univariate		Multivariate Serum VD Level (nmol/L)		Multivariate (VD Deficiency)	
Characteristics	HR (95% CI)	*p*-Value	HR (95% CI)	*p*-Value	HR (95% CI)	*p*-Value
Age at diagnosis, years	1.03 (1.01–1.05)	0.002	1.03 (1.01–1.05)	0.004	1.03 (1.01–1.05)	0.009
Sex, male	2.02 (1.11–3.68)	0.02	1.26 (0.67–2.39)	0.48	1.48 (0.79–2.79)	0.22
Hypertension	1.34 (0.83–2.14)	0.23				
Diabetes	2.39 (1.33–4.32)	0.004	2.21 (1.20–4.06)	0.01	2.19 (1.19–4.04)	0.01
UDCA-incomplete response	1.99 (1.27–3.14)	0.003	2.07 (1.29–3.34)	0.003	2.02 (1.25–3.28)	0.004
BMI ≥ 25 kg/m^2^	0.79 (0.49–1.28)	0.34				
Serum VD level, nmol/L	0.99 (0.98–0.99)	<0.001	0.99 (0.98–0.996)	0.002		
VD deficiency	2.43 (1.51–3.92)	<0.001			1.93 (1.17–3.19)	0.01

Abbreviations: VD—vitamin D; BMI—body mass index; CI—confidence interval; HR—hazard ratio; UDCA—ursodeoxycholic acid. Cox proportional hazard analysis was performed to calculate HRs and *p*-values.

**Table 4:** Risk factors for liver-related outcomes (transplantation and death).

	Univariate		Multivariate Serum VD Level (nmol/L)		Multivariate (VD Deficiency)	
Characteristics	HR (95% CI)	*p*-Value	HR (95% CI)	*p*-Value	HR (95% CI)	*p*-Value
Age at diagnosis, years	0.99 (0.97–1.02)	0.69				
Sex, male	1.71 (0.71–4.12)	0.23				
Hypertension	1.30 (0.65–2.60)	0.47				
Diabetes	2.79 (1.30–6.00)	0.009	2.57 (1.15–5.70)	0.02	2.64 (1.19–5.86)	0.02
Cirrhosis at diagnosis	6.04 (2.66–13.72)	<0.001	4.69 (1.89–11.63)	<0.001	5.32 (2.13–13.30)	<0.001
UDCA-incomplete response	2.64 (1.37–5.10)	0.004	1.95 (0.91–4.20)	0.09	1.98 (0.93–4.23)	0.08
BMI ≥ 25 kg/m^2^	0.56 (0.29–1.10)	0.09	0.80 (0.36–1.75)	0.57	0.75 (0.35–1.59)	0.45
Serum VD level, nmol/L	0.97 (0.96–0.98)	<0.001	0.98 (0.97–0.99)	<0.001		
VD deficiency	5.38 (2.76–10.47)	<0.001			3.33 (1.57–7.07)	0.002

Abbreviations: VD—vitamin D; BMI—body mass index; CI—confidence interval; HR—hazard ratio; UDCA—ursodeoxycholic acid. Cox proportional hazard analysis was performed to calculate HRs and *p*-values.

## Data Availability

The datasets generated and analyzed during the current study are not publicly available but are available from the corresponding author on reasonable request.
